# Driving Immune Responses in the Ovarian Tumor Microenvironment

**DOI:** 10.3389/fonc.2020.604084

**Published:** 2021-01-15

**Authors:** Franklin Ning, Christopher B. Cole, Christina M. Annunziata

**Affiliations:** Translational Genomics Section, Women’s Malignancies Branch, National Cancer Institute (NCI), National Institutes of Health (NIH), Bethesda, MD, United States

**Keywords:** ovarian cancer, tumor microenvironment, innate immunity, adaptive immunity, cancer therapeutics

## Abstract

Ovarian cancer is the leading cause of death among gynecological neoplasms, with an estimated 14,000 deaths in 2019. First-line treatment options center around a taxane and platinum-based chemotherapy regimen. However, many patients often have recurrence due to late stage diagnoses and acquired chemo-resistance. Recent approvals for bevacizumab and poly (ADP-ribose) polymerase inhibitors have improved treatment options but effective treatments are still limited in the recurrent setting. Immunotherapy has seen significant success in hematological and solid malignancies. However, effectiveness has been limited in ovarian cancer. This may be due to a highly immunosuppressive tumor microenvironment and a lack of tumor-specific antigens. Certain immune cell subsets, such as regulatory T cells and tumor-associated macrophages, have been implicated in ovarian cancer. Consequently, therapies augmenting the immune response, such as immune checkpoint inhibitors and dendritic cell vaccines, may be unable to properly enact their effector functions. A better understanding of the various interactions among immune cell subsets in the peritoneal microenvironment is necessary to develop efficacious therapies. This review will discuss various cell subsets in the ovarian tumor microenvironment, current immunotherapy modalities to target or augment these immune subsets, and treatment challenges.

## Introduction

Ovarian cancer presents a unique tumor microenvironment (TME) with its predilection to metastasize in the peritoneal cavity and generate malignant ascites. The cancer spreads by direct shedding into the ascites and movement throughout the peritoneal cavity. Common sites of tumor deposits are on the mesenteric and serosal surfaces of the abdominal organs. The immune microenvironment in this location is characterized by interactions among the tumor cells, myeloid and lymphoid immune cells, as well as fibroblasts and adipocytes in the peritoneum that promote tumor growth. Growth factors, such as fibroblast growth factor and vascular endothelial growth factor (VEGF), promote angiogenesis and direct fibroblast differentiation towards cancer-associated fibroblasts that promote metastases (1). Adipocytes in the omentum can also provide energy for tumor growth and metastases (2). Little is known about how these cells interact with immune cells and if they promote immunosuppression. Additional information is therefore needed about cellular interactions and trafficking in the peritoneal TME in order to better develop immunotherapies for ovarian cancer.

**Figure 1:**
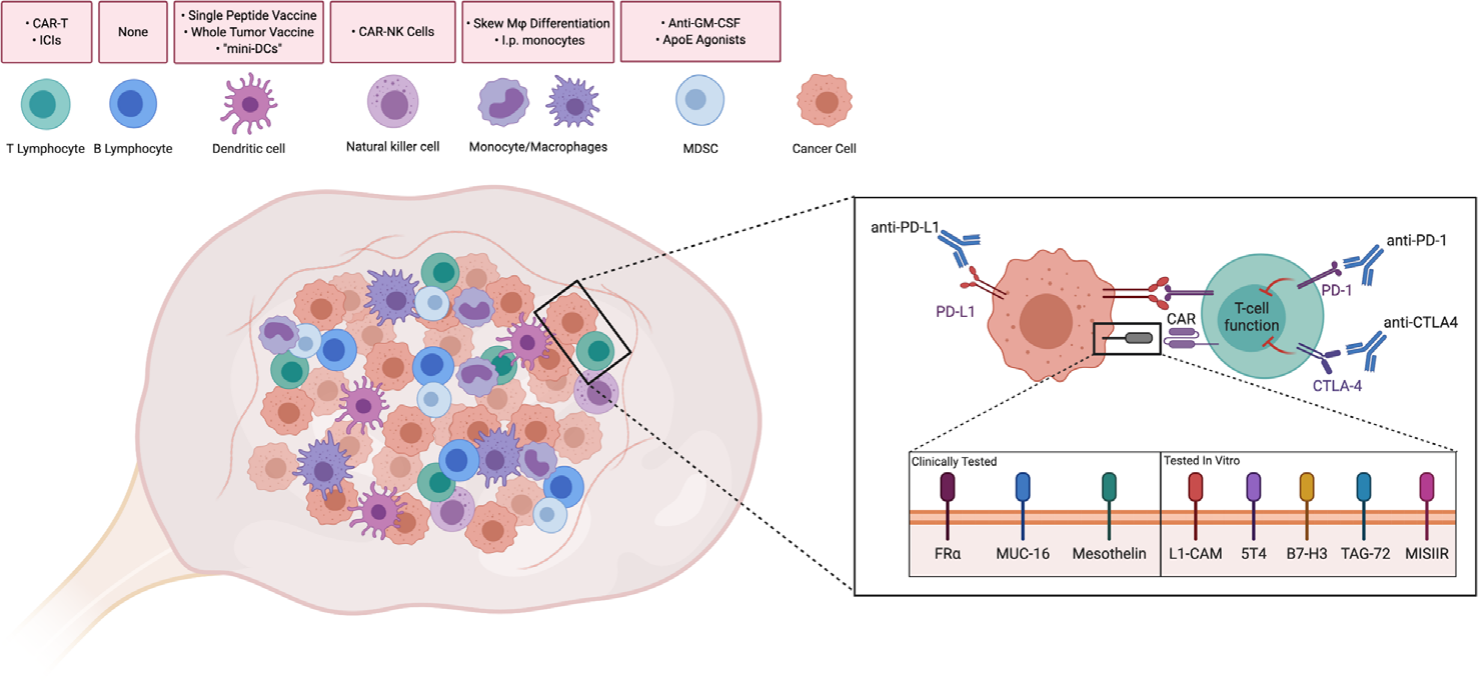
Driving Immune Responses in the Ovarian Tumor Microenvironment. Immune cells are present intratumorally and in the ovarian tumor microenvironment. Strategies discussed throughout the paper have been summarized above the corresponding cell type. Attempts to improve T cell functionality in the ovarian TME include Immune checkpoint inhibitors, such as anti-PD-1, anti-PD-L1, and anti-CTLA4, which have been used in clinical trials to reduce inhibitory signaling. Similarly, T cells with chimeric antigen receptors (CAR-Ts) against folate receptor-alpha (FRα), MUC-16, and mesothelin have been tested in clinical trials in order to recognize tumor-associated antigens. CAR-Ts have been tested *in vitro* against novel antigens. CAR-T: Chimeric antigen receptor T-cell. ICI, Immune checkpoint inhibitor; CAR-NK, Chimeric antigen receptor-Natural Killer cell; Mφ, Macrophage; I.p., Intraperitoneal; GM-CSF, Granulocyte-monocyte colony stimulating factor; ApoE, ApolipoproteinE; MDSC, Myeloid-derived suppressor cell. Created with Biorender.com.

Advances in immunotherapy, such as immune checkpoint inhibitors and chimeric antigen-receptor T cells (CAR-T), have demonstrated efficacy in various cancers. However, performance in ovarian cancer patients has remained poor. Multiple studies have demonstrated that the highly immunosuppressive TME and low mutational burden of ovarian cancer is a barrier to effective treatment (3). Inhibitiory cells in the TME, such as regulatory T and B cells, myeloid-derived suppressor cells (MDSCs), and tumor-associated macrophages (TAMs) inevitably contribute to tumor growth through a milieu of inhibitory effects (4–7). In this review, we discuss key subsets of adaptive and innate immunity that play a role in the ovarian TME and current efforts to target or augment these populations (**Figure 1**).

## Adaptive Immunity

### Tumor-Infiltrating T Lymphocytes

T cells play a significant role in anti-tumor processes by recognizing tumor neoantigens and facilitating and directly inducing apoptosis of tumor cells. CD3^+^ tumor-infiltrating T lymphocytes (TILs) were shown to be correlated with improved clinical outcome in ovarian cancer (8). Of 186 tumor samples, 102 samples were identified to have CD3^+^ cells within the tumor and 72 did not have any. Between these two groups, the 5-year overall survival (OS) for patients with TILs was 38% while those without TILs was 4.5%, suggesting a beneficial effect of TILs in women treated with standard chemotherapy. Interestingly, the absence of TILs correlated with increased levels of VEGF.

TILs, CD3^+^, can be further divided into CD4^+^ and CD8^+^ cells. In brief, CD4+ T cells, also known as helper T cells, recognize MHC class II and shape the adaptive immune response while CD8+ T, also known as cytotoxic T cells, recognize MHC class I and mediate direct killing Studies looking at patient survival have shown increased CD8+ T cells within the tumor predict better prognoses (9). An increase in intra-tumoral CD4+ T cells have also shown to be correlated with increased survival (10).

### ACT/CAR-T

To boost the tumor-specific T cell response, adoptive cell therapy (ACT) has been used to increase the number of T cells that can recognize a tumor-associated antigen (TAA). ACT requires apheresis of a patient’s T cells and expanding them *ex vivo* to suitable levels after stimulation with lysed tumor cells. Recent advances in autologous therapy now include genetically modifying the T-cell receptor (TCR) or generating chimeric antigen receptor T-cells (CAR-T) to engineer a stronger, more precise, immune response to pre-determined tumor neoantigens (11).

Briefly, CAR-T cells are T cells that have been transfected to express a transmembrane protein with 1) a single chain fraction variable, also known as the antigen-recognizing domain, and 2) a TCR zeta chain, allowing for intracellular signaling. Since then, new generations have modified the CAR for improved immune responses. Second generation CAR-Ts added in either CD28 or 4-1BB as a costimulatory gene, third generation CAR-Ts allowed for two downstream signaling domains and the possibility of using OX40, and fourth generation CAR-Ts further improved effector functions by giving the receptor the ability to induce cytokines, such as IL-12 (12).

ACT and CAR-T have shown great promise in hematological tumors. However, they have demonstrated poor efficacy in solid tumors (13, 14). Part of this has been attributed to a lack of tumor specific antigens, a highly immunosuppressive TME, and a lack of persistence in the tumor. Some CAR-Ts that have made it to Phase 1 clinical trials for ovarian cancer include those targeting folate receptor, MUC-16, and mesothelin (**Figure 1**). Other examples in pre-clinical testing are also discussed.

#### Folate Receptor 

Folate receptor-α (FRα) is one of a number of high affinity receptors that facilitates the uptake of folate into the cell (15). While it is rarely found in normal tissues, its overexpression has been identified in multiple malignancies, including ovarian cancer (16). When overexpressed in ovarian cancer, FRα has been correlated with a poor response to chemotherapy (17–20). Interestingly, correlation with survival has been inconclusive. Studies on FR overexpression have ranged from negatively prognostic to having no impact on survival to even an improvement in survival (19–22). Additional studies will be needed to determine the prognostic value of FRα.

In the first CAR-T treatment against FR, 14 patients with recurrent epithelial FR^+^ ovarian cancer were enrolled. Cohort 1 was given interleukin-2 (IL-2) and generic T cells transfected with a first generation anti-FR CAR while Cohort 2 was given T cells that were endogenously specific to FR (23). Cohort 1 was given 3 × 10^9^ T cells, with possible dose escalation to 1 × 10^10^ and 3 to 5 × 10^10^ cells, with a dose of 720,000 IU/kg IL-2 each cycle. Cohort 2 was given 2 × 10^9^ to 4 × 10^9^ T cells. Most common Grade 1-2 drug-related side effects in Cohort 1 were fatigue and nausea compared to erythema at the site of injection for Cohort 2. Cohort 1 also experienced some Grade 3–4 side-effects, such as hypotension and dyspnea, that were attributed to the addition of IL-2. Unfortunately, no reduction in tumor burden or improvement in survival was seen in either cohort. A noted issue was the lack of persistence of these improved autologous T cells in circulation.

As a means of increasing persistence, investigators have added the co-stimulatory molecule CD137, or 4-1BB, to the CAR in order to improve cytokine secretion and the antitumor response *in vivo* (24). Intravenous (i.v.) injection of these CAR-Ts into NSG mice, inoculated subcutaneously with FR^+^ SKOV3, showed improved antitumor effects and reduced tumor volume. These T cells also showed improved persistence in circulating blood. Upon waiting 30 days for metastases in their model, only mice treated with the CAR-T cells were devoid of malignant ascites.

Although a thorough discussion is outside the scope of this review, a number of monoclonal antibodies (mAbs) have also been designed to target FR expression in ovarian cancer. In brief, farletuzumab was evaluated in a Phase 3 clinical trial, but did not reach the primary endpoint of PFS. Mirvetuximab soravtansine, an anti-FR coupled to tubulin-targeting agent DM4, was shown to be well-tolerated when given as a monotherapy or with bevacizumab (25–27).

#### MUC-16

Cancer antigen 125 (CA-125) is cleaved from the cell surface and is commonly used as a circulating serum marker for ovarian cancer relapse. MUC-16 is the remnant of the protein that is retained on the cancer cell membrane after cleavage. It is known that there is some overlap in MUC-16 expression with derivates of the fetal coelomic epithelia, such as the uterus, fallopian tubes, and the trachea (28).

Chekmosova et al. demonstrated that second generation CAR-Ts against MUC-16 were able to lyse MUC-16^+^ cells *in vitro* (29). These CAR-T cells were subsequently injected into SCID mice, inoculated i.p. or i.v. with MUC-16^+^ OVCAR3, and showed significant survival improvement when compared to untreated mice or those given anti-CD19 CAR-Ts. Later improvement to MUC-16 specific CAR-T cells included the ability to secrete IL-12 and an “elimination” gene to improve immune signaling and minimalize off-target effects (30). This “elimination” gene is a truncated portion of epidermal growth factor receptor (EGFR) that does not signal, but retains the ability to be bound by cetuximab and induce antibody-dependent cellular cytotoxicity or complement-mediated cytotoxicity against cells expressing this chimeric receptor. Mice inoculated i.p. with SKOV3 and treated with this novel CAR-T showed enhanced survival compared to CAR-Ts without the IL-12 domain. Interestingly, i.p. administration was found to be more effective than an i.v. route. A phase 1 clinical trial was proposed to test its efficacy in recurrent platinum-resistant ovarian cancer (31).

Similar to FR, mAbs have also been developed against CA-125. One of the leading antibodies, oregovomab, enhanced anti-cancer activity when given with carboplatin and paclitaxel, although oregovomab did not show clinical efficacy when treating patients with ovarian cancer as a monotherapy (32, 33). The combination significantly improved PFS to 41.8 months, when given front-line, compared to 12.2 months for patients given standard carboplatin-paclitaxel (34). Interestingly, patients who had better survival outcomes had lower levels of HLA-DR-CD14+ MDSCs and a lower neutrophil-and-monocyte to lymphocyte ratio (35).

#### Mesothelin

Mesothelin is a surface antigen found overexpressed on malignant mesothelioma as well as pancreatic, ovarian, and lung cancers (36). However, it is also found expressed at low levels on other mesothelial surfaces, such as the pleura, pericardium, and peritoneum.

In ovarian cancer, a mesothelin-specific CAR-T was developed using mRNA transfection in order to minimalize off-target toxicity due to CAR-T persistence (37, 38). Five patients with ovarian cancer were inoculated i.v. with second-generation CARs against mesothelin in a phase 1 clinical trial (39). Up to 1 to 3*10^8^/m^2^ CAR-T cells were given with or without lymphodepletion by 1.5 g/m^2^ cyclophosphamide. Only one patient demonstrated a sizable reduction in tumor burden, but did not qualify as a partial reduction per formal criteria for objective response. This may have been due to a lack of persistence of CAR-Ts by day 28 in most patients and the single-chain variable fraction being murine-based. There were also no significant changes in cytokine levels during the first month post-infusion. The most common low-grade adverse events (AEs) included fatigue, nausea, and emesis, while development of Grade 3 ascites was the most common high-grade AE. A similar Phase 1 trial is evaluating MCY-M11, a mRNA-based anti-mesothelin therapy, when given intraperitoneally (i.p.) for platinum-resistant OC patients, with or without cyclophosphamide (NCT03608618). Another clinical trial evaluating anti-mesothelin CAR-T cells, but generated using a lentiviral delivery system, is ongoing (NCT03054298).

Recently, Hassan et al. published the first in-human clinical trial evaluating anetumab, an anti-mesothelin mAb in a Phase 1 clinical trial (40). Sixty-four patients were enrolled with ovarian cancer, and of those, they noted 1 CR, 4 PRs, and 29 patients with SD. Interestingly, all patients who responded had high mesothelin expression, defined as ≥60% by immunohistochemistry staining. A Phase 1b clinical trial is currently ongoing (NCT02751918).

#### Other Tumor-Associated Antigens

Other surface antigens have been identified in pre-clinical studies as possible neoantigens with anti-tumor activity and high specificity for ovarian cancer. Hong et al. showed that L1-CAM was overexpressed in a wide panel of ovarian samples (41). Administration of second-generation anti-L1-CAM CAR-Ts, to NSG mice, inoculated i.p. with SK-OV3, improved median survival time to 104.5 days when compared to mock (50 days) or anti-CD19 CAR-T cells (56.5 days). Similar tissue expression, *in vitro*, and *in vivo* studies can be seen with 5T4, B7-H3, TAG-72, and MISIIR (42–45). Interestingly, Du et al. demonstrated the addition of 4-1BB to the anti-B7-H3 CAR mediated lower expression of PD-1 in transfected CD8^+^ T cells, possibly enabling them to better enact effector functions. In this pre-clinical study, i.p. administration of CAR-T cells was shown to have improved survival benefit when compared to an i.v. route.

### Immune Checkpoint Inhibitors

Other mechanisms of promoting immune cell activity are immune checkpoint inhibitors that target surface proteins such as cytotoxic T-lymphocyte-associated protein 4 (CTLA4), programmed cell death protein 1 (PD-1), and its ligand (PD-L1; **Figure 1**). These proteins normally function to prevent autoimmunity, but their upregulation on a tumor prevents an appropriate immune response. CTLA-4 competes with B7 (CD80) in binding to CD28 on T cells, inducing a suppressive phenotype rather than activating. PD-1 and PD-L1 belong to the CD28 and B7 family of receptors on T cells, respectively, and have been shown to induce a suppressive phenotype in peripheral tissues.

Drugs targeting CTLA-4, such as ipilimumab, have shown great success in other cancers. In unresectable Grade 3 and 4 melanoma, ipilimumab improved 1-year survival from 25% to 46% when used in combination with a gp-100 peptide vaccine, granting it FDA approval in March 2011. In a phase 1 clinical trial with nine Stage IV ovarian cancer patients, three instances of stable disease (SD) were observed (46). Multiple toxicities occurred, including two cases of Grade 3 inflammation in the GI tract, one case of Sweet’s syndrome, and multiple dermatological reactions. A phase 2 clinical trial was later done in patients with recurrent platinum-sensitive ovarian cancer (NCT01611558). Patients received 10 mg/kg of ipilimumab once every 3 weeks for four doses then once every 12 weeks. The overall response rate (ORR) was low at 10.3%. 18 patients experienced drug-related severe AEs, the most common being small intestinal obstruction, diarrhea, pneumonitis, and adrenal insufficiency.

Trials combining ipilimumab with other treatment regiments have also been conducted. Recently, Zamarin et al. published a Phase 2 trial comparing nivolumab to nivolumab and ipilimumab for recurrent ovarian cancer. One hundred patients were either given 3 mg/kg nivolumab every 2 weeks or 3 mg/kg nivolumab plus 1 mg/kg ipilimumab every 3 weeks (47). In the nivolumab monotherapy group, 3 complete responses (CRs), 3 partial responses (PRs), and 14 patients with SD were noted compared to the 3 CRs, 16 PRs, and 20 patients with SD in the combination group. Median progression-free survival (PFS) was 2 and 3.9 months while median OS was 21.8 and 28.1 months for the monotherapy and combination therapy, respectively. There were no treatment-related deaths with the most common grade 3 or higher AEs being asymptomatic elevation of pancreatic and liver enzymes, anemia, colitis, and diarrhea. A number of other ongoing trials are evaluating anti-CTLA-4 antibodies in combination with chemotherapy, poly (ADP-ribose) polymerase inhibitors (PARPi), and other immunotherapies (**Table 1**).

**Table 1:** Ongoing clinical trials of immune checkpoint inhibitors in women with ovarian cancer.

Intervention	NCT/Author	Phase	Enrollment	Primary Endpoint
**Anti-CTLA4**				
Tremelimumab + Olaparib	NCT02571725	1/2	~50 patients with BRCA1/2-mutant ROC	RP2D, ORR
Tremelimumab + Olaparib	NCT04034927	2	~170 RPS OC	
**Anti-PD-1**				
Nivolumab ± Ipilimumab	NCT03355976	2	~62 patients with ovarian or renal cell carcinoma	ORR
Intraperitoneal Nivolumab ± Ipiliumumab	NCT03508570	1b	~48 patients with recurrent/high-grade gynecologic cancer with peritoneal metastases	RP2D
Nivolumab ± Ipiliumumab + CarboTaxol	NCT03245892	1	~40 patients with High Grade Serous Ovarian, Fallopian Tube, or Primary Peritoneal Cancer	DLT
Nivolumab + Bevacizumab ± Rucaparib	NCT02873962	2	~76 patients with ROC	ORR
Nivolumab + Poly-ICIC (a viral mimic)	NCT04024878	1	~30 patients with OC	Safety and Activity
Nivolumab + WT1 vaccine	NCT02737787	1	~11 patients with ROC	DLT
Durvalumab ± Tremelimumab + CarboTaxol	NCT03249142	1/2	~40 patients with Ovarian, Fallopian Tube or Primary Peritoneal Adenocarcinoma	DLT
Durvalumab + Tremelimumab + CarboTaxol	NCT03899610	2	~24 patients with treatment-naïve clinical stage IIIC/IV ovarian cancer	PFS
Sequential vs. combination Durvalumab + Tremelimumab	NCT03026062	2	~100 patients with RPR OC	irPFS
Olaparib + Tremelimumab + Durvalumab	NCT02953457	1/2	~36 patients BRCA1/2-mutant ROC	DLT and PFS
**Anti-PD-L1**				
Avelumab ± PLD	NCT02580058	3	566 patients with RPR OC	OS and PFS

RP2D, Recommended phase 2 dose; ORR, Overall response rate; RPS, recurrent platinum-sensitive; OC, ovarian cancer; PFS, Progression free survival; DLT, dose-limiting toxicity; irPFS, immune-related progression free survival; CarboTaxol, carboplatin and paclitaxel; ROC, recurrent ovarian cancer; PLD, pegylated liposomal doxorubicin; OS, overall survival.

Anti-PD-1 therapy has also had some success in treating ovarian cancer. In a phase two trial, 20 patients with platinum-resistant recurrent ovarian cancer were treated with either 1 or 3 mg/kg single-dose nivolumab every 2 weeks for one year (48). In the 1 mg/kg cohort, two patients had a CR and four had SD while in the 3 mg/kg cohort, one patient had a PR while two had SD. Median OS was 20 months and PFS was 3.5 months. Low-grade AEs included mild fever, rash, arthralgia, elevated liver function tests (LFTs), and lymphocytopenia. Two patients experienced Grade 3 disorientation and gait disorder or Grade 3 fever and a deep vein thrombosis, respectively. Notably, two patients developed a PR to maintenance chemotherapy post-treatment with nivolumab (49). An open-label, randomized clinical trial is ongoing in Japan.

One Phase 2 study has been published evaluating the combination of nivolumab and bevacizumab in relapsed ovarian cancer (50). Thirty-eight women with relapsed OC were enrolled and treated with 10 mg/kg bevacizumab and 240 mg nivolumab once every 2 weeks. In platinum-sensitive patients, there were eight PRs and nine patients with SD compared to 3 PRs and 10 patients with SD in the platinum-resistant group. Median PFS was 12.1 months and 7.7 months for platinum-sensitive and -resistant patients, respectively. Grade 3 AEs reported were hypertension, myalgia, arthralgia, and elevations in LFTs and serum amylase. Two patients experienced Grade 4 increases in serum lipase levels as a result of treatment. Like anti-CTLA-4 compounds, several ongoing trials are evaluating combinations of nivolumab with chemotherapy, PARPi, or vaccines (**Table 1**).

Pembrolizumab, another anti-PD-1 agent, has also been evaluated as a single agent. In a phase 1b trial, 26 patients with advanced metastatic ovarian cancer received 10 mg/kg every 2 weeks for up to two years (51). ORR was documented at 11.5%, with one CR and two PRs, and seven patients experienced SD. Median PFS was 1.9 months while OS was 13.8 months. Drug-related AEs occurred in 19 patients with one patient experiencing Grade 3 increases in transaminase levels. Eight immune-related AEs occurred where the only Grade 3 AE was pancreatitis. Later, Keynote-100 looked at pembrolizumab in 396 patients with advanced recurrent ovarian cancer. Two cohorts were formed based on prior lines of treatment (52). Cohort A (285 patients) had one to three lines of prior treatment while cohort B (91 patients) had four to six. Patients received 200 mg IV every 3 weeks. ORR in cohorts A and B was 7.4% and 9.9%, respectively. Median PFS was 2.1 months for both cohorts while median OS was not reached in cohort A and 17.6 months in cohort B. The most common low-grade treatment-related AE was fatigue while the most common immune-related AE was hypothyroidism. The most common Grade 3 immune-related AEs were skin reactions and colitis. More severely, two patient deaths were attributed to treatment, one due to hyperaldosteronism and the other to Stevens-Johnson syndrome. These recurring AEs led to the need for early recognition and treatment to prevent devastating complications from immune checkpoint inhibitor therapy (53).

Recently, a single-arm phase I/II trial evaluated niraparib in combination with pembrolizumab in patients with recurrent platinum-resistant OC (54). 62 patients with OC were enrolled between Phase I and II. ORR was 18% with 3 CRs and 8 confirmed PRs. Another 28 patients were noted to have SD. Median PFS was 3.4 months. Most common low-grade AEs were fatigue, nausea, anemia, and constipation while high-grade AEs were noted to be anemia and thrombocytopenia. Extensive ongoing work is exploring combination therapies with pembrolizumab.

Durvalumab, another anti-PD-1 compound, has been tested in combination with PARPi in both Phase I and II trials. In a Phase 1 trial, seven patients with ovarian cancer, along with one endometrial and triple negative breast cancer, were treated with olaparib and cediranib, a VEGFR1-3 inhibitor (55). ORR was 44% with four partial responses and three patients with SD. Most common low-grade AEs were fatigue, nausea, and increased LFTs. Five patients experienced grade 3 hematological AEs, three with lymphopenia and two with anemia). In a subsequent Phase 2, 35 OC patients were enrolled in a single-center study (56). ORR was noted to be 14% with five patients achieving a partial response while 20 patients had SD. Overall, median PFS was noted to be 3.9 months. Like the Phase 1, hematologic toxicity, mainly anemia, was the most common high-grade AE, affecting eleven patients. The study also found significant increases in VEGFR3 were correlated with worse PFS. Of note, when evaluating durvalumab and olaparib in PARPi-naïve patients with platinum-sensitive and mutated BRCA in a Phase II trial, a 63% RR was noted, with six patients achieving a CR and fourteen achieving PRs (57). The most common grade three or higher AEs were anemia, increased lipase and amylase, and neutropenia.

Avelumab, an anti-PD-L1 therapy, though not directly inhibiting the suppression of T cells, plays a role in preventing the inactivation of T cells. In a phase 1b trial, 125 ovarian cancer patients with Stage III or IV disease received 10 mg/kg avelumab every 2 weeks (58). Confirmed objective response was seen in only twelve patients with one CR and eleven PRs. Median OS was 11.2 months. The most common treatment-related AEs were fatigue, diarrhea, and nausea while the most common high-grade was an increase in lipase-levels. Low grade immune-related AEs mainly were hypothyroidism while three patients separately experienced high-grade colitis, type 2 diabetes, or myositis. Ongoing clinical trials are evaluating avelumab with chemotherapy and PARPi (**Table 1**).

### Regulatory T Cells

Discovered in 1995, Tregs were originally shown to be involved in immune homeostasis, preventing an over-activation of the immune system towards self (59). They have been characterized to express CD4, CD25, and, most notably, FoxP3 (4). Tregs enact their function by suppressing activation of immune cells, inducing cell death of effector cells, and secreting anti-inflammatory cytokines, such as TGF-beta and IL-10 (60). Since their discovery, Tregs have been shown to be involved in a number of disease processes, including cancer.

In ovarian cancer, Tregs have been shown to be an indicator of poor prognosis. Curiel et al. showed that in 104 patients, increased numbers of intratumoral Tregs predicted poor survival (61). Absolute Treg counts may overlook certain cellular interactions, because in a study of 117 patients, intraepithelial Treg counts did not correlate with a significant difference in survival (9). Instead, increased ratios of CD8^+^ T cells to CD4^+^CD25^+^FoxP3^+^ T cells were shown to significantly associate with improved survival. Later studies also showed that a high CD8^+^/Treg ratio as well as CD4^+^/Treg ratio were associated with better survival outcomes (62).

Treg function can also be influenced by the ovarian TME. TAMs and tumor cells have been shown to increase levels of CCL22, which aid in Treg recruitment to the ovarian TME (61). In a set of 75 ovarian cancer patients, CCL22 levels were shown to be elevated in the peritoneal fluid, possibly contributing to Treg recruitment and cancer progression (63). Tregs have also been shown to be highly activated when found intratumorally in ovarian cancer. CD45RA^-^FoxP3^hi^ effector Tregs expressed significantly higher levels of 4-1BB, ICOS, OX40, and CTLA4 compared to CD45RA^-^FoxP3^lo^ effector T cells (64). Tregs expressing high PD-1 and 4-1BB were subsequently both more responsive to stimulation by anti-CD3/anti-CD28 and were able to better suppress T cells *in vitro*.

One method of reducing Treg effector functions is to directly deplete Tregs. Low-dose cyclophosphamide has been tested for use in conjunction with cancer vaccines due to its ability to deplete FoxP3 Tregs (65). However, Tregs treated with low-dose cyclophosphamide were unable to suppress the proliferation of CD4+ and CD8+ T cells *in vitro*. When tested in ovarian cancer, a combination therapy of low-dose cyclophosphamide with a p53-SLP vaccine did not directly suppress either Treg counts or functionality (66). However, overall T cell counts were higher and persisted longer in the combination group when compared to vaccination alone. Similarly, a phase 1/2 clinical trial found a single i.v. dose of cyclophosphamide had no effect on circulating Tregs (67).

### Regulatory B Cells

In ovarian cancer, there is evidence infiltrating B cells can be either good or bad prognostic indicators. Milne et al. found that intraepithelial CD20+ B cells, when present in patients optimally debulked from high-grade serous OC, positively correlated with disease-specific survival (68). Patients who had residual disease or another histological subtype, however, did not demonstrate any significant survival benefit with infiltrating CD20+ B cells. In a follow-up study, tumor-infiltrating CD20+ cells were found to have responded to antigen, having undergone class switching, somatic hypermutation, and clonal expansion (69). These CD20+ B cells, when found co-localized with CD8+ T cells in tumor, also correlated with improved patient survival compared to tumor-infiltrating T cells alone. In an independent study, Santoiemma et al. also found tumor-infiltrating CD20+ B cells to positively correlate with OS (70). Later, these CD20^+^ B cells, in addition to CD138^+^ plasma cells and CD4^+^ TILs, were found to co-localize with CD8^+^ T cells in tertiary lymphoid structures and indicate better prognosis (71). Interestingly, Kroeger et al. also found tumor-infiltrating plasma cells, in high-grade serous ovarian cancer, to express IgG and CXCR3, the latter being normally expressed under immuno-stimulatory environments (72).

Contrary to this finding, Lundgren et al. found CD138+ plasma cells to positively correlate with tumor grade and negatively with OS (73). Furthermore, they found CD20+ B cells only correlated with tumor grade and had no significant correlation with survival. Other studies have also shown infiltrating B cells to be detrimental. Yang et al. showed that high levels of CD19+ B cells in the omentum correlated with poor survival (74). Ultimately, additional phenotype characterization of tumor-infiltrating B cells needs to be completed to determine their impact in the TME and patient outcomes.

Recently, a subset of B cells known as B regulatory cells has been found in ovarian cancer patients (75). More specifically, IL-10^+^ B cells were increased in ascites compared to peripheral blood. These B cells were inversely correlated with the number of CD8^+^ T cells in ascites and positively correlated with FoxP3^+^CD4^+^ T cells. *Ex vivo* studies showed these B cells were capable of suppressing IFN gamma secretion by T cells even under stimulation by anti-CD28. Finally, accumulation of these cells in the ascites correlated with more late-stage and aggressive disease. In models of spontaneous ovarian cancer, increased CD25+ pre-B-like cells were found intratumorally (76). These pre-B-like cells were shown in breast cancer models to develop into tBregs (CD19+ CD25^Hi^CD69^Hi^) and promote metastases. There are no modalities currently available that specifically target Bregs. Additional research is necessary to determine which markers best define this subset and how they function in ovarian cancer.

## Innate Immunity

Despite recent advances in the use of checkpoint inhibitors and augmenting the T cell response, attempts at augmenting adaptive immunity have been unsuccessful in treating ovarian cancer patients. Recent efforts have shifted to include exploiting the innate immune response (77). Myeloid cells of the innate immune system, such as monocytes, classical macrophages, natural killer cells, and dendritic cells, all play key roles in promoting an effective adaptive response; the lack of accounting for these interactions may be where current immunotherapies fall short. Alternatively, cell types such as TAMs and MDSCs induce a highly immuno-suppressive TME and may be targets of future therapeutic strategies (6, 7).

### Dendritic Cells 

As the main mediator of responses between the innate and adaptive immune response, conventional human DCs (cDCs, CD141+, or CD1c+), are critical for the adaptive response by up-taking antigen and skewing helper T cell differentiation (78). However, cancer cells subvert proper antigen presentation by down-regulating MHC, reducing TAAs on their surface, and can be suppressed by numerous cytokines (79). DCs can also induce T cell suppression themselves through PD-1 and CD277 in the ovarian cancer TME (80, 81). Human plasmacytoid dendritic cells (pDCs, CD303+) increase immunosuppression in the ovarian cancer TME through upregulation of Tregs (82, 83). Furthermore, tumor-associated pDCs have been correlated with poor prognosis and early relapse for ovarian cancer patients, possibly due to their influence on CD4+ T cells to produce increased IL-10 (84).

Multiple clinical trials have looked at the benefit of utilizing DCs, obtained through leukapheresis or derived from monocytes, that are pulsed with specific antigens as immunotherapy. In one trial, autologous DCs were pulsed with Her2/neu, human telomerase reverse transcriptase, and pan-DR epitope with or without a single-dose of cyclophosphamide. Five of eleven patients had no evidence of disease at the time of publication with only one patient dying of disease within 36 months after the initial vaccination (67). No grade 3 or 4 treatment-related AEs were reported. Two studies introduced IL-2 in conjunction with DC treatment. Rahma et al. evaluated the optimal mechanism of peptide delivery to DCs, with 6 OC patients receiving DCs pulsed *ex vivo* with wild-type p53 peptide 264-272 (85). PFS and median OS was found to be 8.7 and 29.6 months, respectively, comparable to those patients receiving subcutaneous injections of solely peptide. Notably, all Grade 3 or 4 AEs occurred during cycles of IL-2 administration—the most common being fatigue, lymphopenia, and elevated liver enzymes. IL-2 administration also led to increases in Tregs. In another study by Baek et al., 10 patients with minimal residual disease were treated with DCs pulsed with keyhole limpet haemocyanin (KLH) and IL-2 (86). KLH has previously been used as a surrogate marker for DC vaccination. Three patients underwent complete remission with the most common AEs being flu-like symptoms, attributed to IL-2 administration. Contrastingly, this study found treatment decreased CD4+CD25+ T cells, albeit they did not characterize FoxP3 expression. A recent Phase I/II study enrolled three ovarian cancer patients and treated them with DCs pulsed with Wilms’ tumor protein 1 (87). One patient reached SD by RECIST criteria while the other two had progressive disease. No Grade 3 or greater AEs were reported.

Alternatively, DCs could be pulsed with whole tumor lysate as a means of eliciting a response to a variety of neoantigens as opposed to a single one. In a phase I study with six ovarian cancer patients, autologous DCs were pulsed with autologous tumor lysate and (KLH) (84). No grade 2 or higher AEs reported. Most common Grade 1 AEs included pain, fatigue, nausea, and abdominal pain. In another clinical study, 25 immunotherapy-naïve OC patients were treated with either a) intranodal injections of DCs pulsed with oxidized whole tumor lysate, b) whole-tumor lysate pulsed DCs with bevacizumab or c) the prior combination with cyclophosphamide (88). No toxicities greater than Grade 2 were reported due to the treatment. The most common Grade 1 AEs overall were pain, fatigue, nausea and abdominal pain. There were two PRs and thirteen patients had SD. Of note, patients without intratumoral T cells reactive for autologous tumor had poorer outcomes, again suggesting the success of DC vaccinations relies on the ability to generate a specific T cell response. Interestingly, the addition of cyclophosphamide improved both immune response, as measured by IFN-gamma release, and ultimately patient survival.

But despite some success of these DC vaccines, limitations include the ability to generate a consistent immuno-stimulatory effect and the difficulty of vaccine production (89). In an attempt to generate a more efficient T-cell response in a preclinical setting, Mirandola et al. treated DCs infected with a recombinant adeno-associated virus (rAAV) containing cancer/testis antigen mSP17 with a p38 MAPK inhibitor (90). A p38 inhibitor was used due to previous studies showing its use improving monocyte-derived DC survival and decreasing Treg production (91, 92). Murine DCs were infected with rAAV-mSP17 and treated with a p38 MAPK inhibitor. Survival analyses showed that when C57BL/6 mice, injected i.p. with 1 × 10^6^ ID8 cells, were treated with DCs plus p38 inhibitor, 95% of mice survived up to 300 days, as compared to those receiving solely DCs surviving up to 98 days. Furthermore, the addition of p38 inhibitor increased lymphocyte trafficking to the tumor. When using human DCs, the addition of p38 inhibitor significantly decreased PD-L1 expression, reversing one contributor to the highly immunosuppressive TME. In a separate attempt to alternatively produce DCs for use in OC patients, Cheng et al. developed “mini DCs” through the use of cell membrane coating nanotechnology, i.e., fusing cell membranes onto synthetic polymer cores (93). To traffic tumor antigens to the membrane prior to fusion, ID8 murine cells were lysed and pulsed onto bone marrow-derived DCs. Additionally, IL-2 was loaded into the nanoparticle prior to emulsion. Mice inoculated with ID8 cells subcutaneously showed significant growth reduction when treated with the mini DCs compared to normal DCs and empty nanoparticles. Increased CD8+ T cell infiltration and decreased Tregs were also observed intratumorally in the mini DC treated mice. When evaluating the effect of mini DCs on metastases, mice injected i.p. with ID8 cells and treated with mini DCs had significantly fewer nodules on the peritoneal wall when compared to vehicle or those treated with normal DCs. No changes in body weight or liver and kidney functions were observed in mice treated with mini DCs, indicating good biocompatibility.

### Natural Killer Cells

Natural killer cells, CD56+, have become increasingly popular as an immunotherapy due to their ability to kill without prior sensitization to antigen. Instead, they integrate activating and inhibitory receptors in order to mediate their cytotoxic effect. Receptors, such as killer cell immunoglobulin-like receptors (KIRs) and NKG2A-C that recognize MHC and NKG2D that recognize stress molecules, cooperate to sense “missing self,” “induced self,” or “altered self (94).” Similar to T cells, these cells are able to kill by perforin-granzyme and also by FAS and TRAIL-mediated mechanisms (95). In OC, NK cells have been reported to both be positively and negatively prognostic. One study evaluated the prognostic value of intra-tumoral NK cells in 82 patients with mixed histologies (96). Researchers found patients with only intra-epithelial infiltration of NK cells had an increased OS (106 months) compared to those with only intra-stromal infiltration (58 months); no difference was seen in PFS between these two groups. However, tumor infiltration of CD56+ NK cells did not correlate with prognosis. Additionally, this study evaluated the presence of ULBP2 and MICA/B on patient outcomes—both activating ligands of NKG2D thought of commonly to mark cells for elimination. Interestingly, high levels of ULBP2 on tumor samples was found to indicate a poor prognosis for cancer patients, while MICA/B did not correlate with prognosis. This may be due to high levels of ULBP2 inhibiting proper T cell functioning. Samples from 283 patients with high-grade serous carcinoma were evaluated by immunohistochemistry for NK cell infiltration (97). Median OS in patients with high levels of CD57+ NK cells (≥10 cells/mm^2^) was improved, compared to patients with low levels (<10 cells/mm^2^), 45 vs. 29 months, respectively. Interestingly, higher CD56+ NK cells:lymphocyte fraction in ascites was associated with both a better PFS and OS in 20 OC patients (98). It was noted though that by selecting for patients that had enough ascites, patients with poor prognosis were inadvertently selected. Further studies will be needed to evaluate the importance of intra-tumoral NK cells.

There are multiple clinical trials in progress evaluating the benefits of augmenting NK cell number and function. One Phase I trial is evaluating IP FATE-NK100, a donor-derived NK product compromised from terminally differentiated cells, with IL-2, as a means of promoting NK survival, and lymphodepletion by cyclophosphamide and fludarabine (CyFlu) in women with recurrent OC (NCT03213964). Another Phase I trial is evaluating i.p. NK cells, instead generated from CD34 hematopoietic stem cells in umbilical cord blood, in twelve recurrent OC patients with lymphodepletion by CyFlu (NCT03539406). In an attempt to boost the body’s own NK cells, one Phase II trial is evaluating the use of i.p. as well as subcutaneous (s.q.) IL-15Ra super-agonist, ALT-803, after first-line chemotherapy (NCT03054909). It was previously shown that in OC, ascites-derived NK cells and healthy donor NK cells improved their reactivity when stimulated with IL-15 or ALT-803 (98). One published Phase II study evaluated i.v. NK cells, treated *ex vivo* with IL-2, given post-lymphodepletion by CyFlu in 14 ovarian cancer patients (99). Five patients also received total body irradiation to deplete lymphocytes and allow for NK expansion. Despite four patients reaching PRs and eight having SD, one patient developed a grade 5 toxicity due to tumor lysis syndrome. Other severe AEs, such as passenger lymphocyte syndrome and neutropenia, were attributed to the irradiation.

CARs have been also added to NK cells, as well as NKT cells (CD3+CD56+), in an attempt to utilize these cells. Briefly, NKT cells carry characteristics of both NK and T cells, enabling them to enact cytotoxic killing without prior activation. Utilizing a CAR against FR, Zuo and colleagues showed NKTs with improved cytotoxicity towards FR^+^ PEO1 cells *in vitro* when compared to CAR-Ts by transfecting NKT cells with CARs carrying both the CD28 and 4-1BB co-stimulatory signaling domain, (100). However, the NKT cells performed worse than CAR-T cells in nude mice inoculated subcutaneously with PEO1 cells. NK cells, specifically NK-92, were also used as a surrogate for a third-generation anti-FR CAR (101). Similarly, these NK cells demonstrated cytotoxic effects against SKOV3 *in vitro* and in B-NDG mice inoculated i.p. with SKOV3. One clinical trial utilizing anti-mesothelin NK cells, obtained from peripheral blood mononuclear cells, has been proposed (NCT03692637).

Changes in synthesis methods and receptor signaling may also be beneficial to the success of CAR-NKs. Li et al. created anti-mesothelin CAR-NKs, with additional NKG2D and 2B4 domains, from induced pluripotent stem cells (iPSCs) (102). Advantages to using iPSC include better clonal manipulation of the end product and increased speed of production. In a xenograft model injected i.p. with A1847 cells, treatment with their CAR-NK, along with IL-2 and IL-15, significantly reduced tumor burden and, ultimately, improved survival. When directly compared to a CAR-T with the same receptor, but with 4-1BB and CD28 signaling motifs instead, mice treated with the CAR-NK had less weight loss and pathogenic damage in organs such as the liver and kidney. Furthermore, those treated with CAR-NK also had improved survival when directly compared to those given CAR-T cells. Another group has created a CAR-NKs against glypican-3 (GPC3), with CD28 and 4-1BB signaling motifs, also from iPSCs derived immune cells (103). In NSG mice inoculated i.p. with KOC7c, a GPC3-expressing ovarian cancer cell line, a statistically significant difference in survival was seen when compared to PBS. Klapdor et al. has also developed dual-CAR-NKs, using NK-92, against CD24 and mesothelin with CD28 and 4-1BB signaling motifs (104). CD24 was chosen as a target due to its presence on cancer stem cells and lack thereof on normal tissues; mesothelin has been previously discussed. In A2780 and HEK293T cells previously transfected with CD24, mesothelin, or both, the dual-CAR was able to target both mesothelin-positive cells and CD24-positive cells.

### Monocytes and Macrophages

Monocytes can be classified into three subsets based on CD14 and CD16 expression: classical monocytes (CD14+CD16−), intermediate monocytes (CD14+CD16+), and non-classical monocytes (CD14-CD16+). Upon inflammation, monocytes traffic to the tissue and differentiate on a spectrum ranging from classically activated macrophages, or M1-like, to alternatively activated macrophages, or M2-like. A holistic review on M1 vs. M2 macrophage differentiation is more thoroughly reviewed in (105). In brief, M1-like macrophages, induced by IFN-γ and TNF-α, secrete inflammatory cytokines, such as IL-6 and IL-12, while M2-like macrophages are induced by TGF-β and IL-4/13 and secrete anti-inflammatory cytokines and recruiting Tregs (106). Cancer cells themselves are able to induce a shift towards an M2 phenotype through the secretion of signaling molecules. In OC specifically, CSCs were shown to increase levels of CCL2, COX-2 and PGE-2 as well as activate the PPARγ pathway, all of which correlated with increased polarization towards M2 macrophages (107, 108). Unsurprisingly, a high M1/M2 ratio has been correlated with improved survival in OC patients (109). A meta-analysis also indicated the presence of CD163+ TAMs was correlated with poor prognosis (110). A decreased lymphocyte-to-monocyte ratio (LMR) also indicated both poor overall and PFS in retrospective reviews (111–114).

In order to bolster immunity against cancer, the goal would be to decrease M2-like macrophages and/or increase their M1-like counterparts. Pre-clinical studies have attempted to decrease the prominence of M2 macrophages in the OC TME by interfering with the number of TAMs. Trabectedin, an inhibitor of DNA repair and transcription, was found to activate caspase-8 in monocytes through TRAIL-R1/2, leading to decreases in TAMs (115). Paclitaxel, a microtubule inhibitor currently in use to treat ovarian cancer, was recently found to shift M2 macrophages towards M1 in a TLR4-dependent fashion (116).

In OC, the addition of IFN-α/γ to monocytes was hypothesized to maintain the M1-like phenotype when used as an anti-cancer therapy. Importantly, this combination was shown to significantly reduce tumor burden and improve survival in BALB/c mice inoculated subcutaneously with OVCAR-3 (117). Mice treated with the combination of IFNs and monocytes survived to 170 days when compared to 87 and 81 days for IFNs or monocytes alone, respectively. Intra-tumoral macrophages were identified by CD31 and CD68 staining. Further immunofluorescence (IF) characterization showed that the cells expressed M1 markers IL12, CXCL10 and NOS2, but decreased M2 markers IL-10 and arginase, indicating that the IFN-treated monocytes retained differentiation towards an M1-like phenotype. Extrapolating these findings to other ovarian cancer cell lines, Johnson et al. reinforced the ability of monocytes and IFNs to kill tumor cells synergistically, although sensitivity varied between lines (118). A Phase 1 clinical trial was created to evaluate the intraperitoneal administration of autologous monocytes treated *ex vivo* with pegylated inteferon α-2b and interferon γ-1b (119). Preliminary results showed a well-tolerated treatment with two PRs and four patients with SD (120).

### Myeloid-Derived Suppressor Cell

Compared to the veteran immunosuppressive Tregs (discovered in 1969), myeloid derived suppressor cells (MDSCs) are a relatively “young” subset (121). Poorly-differentiated myeloid cells with the capacity to suppress T cell activation were reported in the 1970s (122), but the term “MDSC” was not coined until 2007 (123), and many questions relating to the origin, classification, and behavior of MDSCs remain unresolved (124).

A comprehensive review of the current understanding of MDSCs have been presented previously (7, 125). Briefly, MDSCs are myeloid derived cells which develop in bone marrow, traffic through peripheral blood to the tumor, and increase during tumor development in response to chemotactic and growth factor signals released by the tumor itself such as G-CSF, GM-CSF, VEGF, and IFN-γ (126, 127). MDSCs are functionally characterized by the ability to suppress T cell activation *ex vivo* (122), and to suppress the ability of immune cells in the TME to mount an antitumor response *in vivo* by mechanisms which are incompletely characterized, but include direct suppression of cytotoxic T cells and NK cells via PDL1/2, promotion of Treg expansion by TGF-beta, CD40L, and Il-10, and promotion of M2-like/TAM development (128). MDSCs can be identified by their expression of specific markers, allowing division into monocytic and granulocytic subtypes according to consensus guidelines, although these classification schemes remain in flux (125). In mice, monocytic MDSCs are defined as CD11b^+^ Ly6C^high^ Ly6G^–^ while granulocytic MDSCs are CD11b^+^ Ly6C^low^ Ly6G^+^. In humans, monocytic MDSCs are HLA-DR^-^CD11b^+^CD33^+^CD14^+^ while granulocytic MDSCs are HLA-DR-CD11b^+^CD33^+^CD15^+^.

The clinical relevance of MDSCs as drivers of ovarian cancer pathogenesis has been demonstrated by correlative studies in humans associating MDSC frequency and phenotype with worse prognosis as well as by experimental manipulations in mice, in which direct ablation of MDSCs can impede tumor development. In patient series, higher MDSC frequency in tumor biopsy (129), in peripheral blood (130), or in ascites (131) correlated with decreased OS or relapse free survival, as did high MDSC-to-dendritic cell ratio in peripheral blood (132). Syngeneic mouse model studies have demonstrated that MDSCs accumulate during the course of tumor development (133) and that ablation of MDSC by clodronate liposomes led to increased survival. Depletion of MDSC by anti-Gr1 antibodies also inhibited tumor growth in mouse and depletion of MDSC led to increased mouse survival whereas adoptive transfer of MDSC from one tumor-bearing mouse to another improved tumor growth (134, 135). Similarly, depletion of MDSCs by anti-GM-CSF therapy reversed anti-VEGF therapy resistance, reducing intra-tumoral MDSCs and increasing CD8^+^ TILs (136).

MDSCs have the capacity to develop into immunosuppressive M2-like macrophages, but they can also differentiate into non-immunosuppressive cell types such as conventional M1 macrophages and dendritic cells. Agents such as all-trans-retinoic acid (ATRA) and epigenetic modifiers such as histone-deacetylase inhibitors (HDACi) have been demonstrated to induce differentiation of MDSC (resulting in functional depletion) in preclinical studies. In ovarian cancer, the phase II trial of histone deacetylase inhibitor entinostat in combination with checkpoint inhibitor avelumab failed to demonstrate an advantage over avelumab alone (137). Differentiation vs depletion of MDSC by the hypomethylating agent azacytidine has been reported in mouse (138). Of note, azacytidine has also been reported to sensitize platinum resistant ovarian cancer cells to platinum by mechanisms that are not entirely understood (139), which has been the inspiration for other trials. Decitabine has been pursued as a platinum sensitizer and reported as having disease activity by CA-125 reduction but not by reduction in tumor size, i.e., RECIST criteria (140). A phase 1 trial of azacytidine and valproic acid, another HDACi, in combination with carboplatin in patients with platinum-refractory solid tumors was reported in abstract form as displaying disease activity but with a very high (78%) rate of grade 3–4 toxicity (141).

Blocking of MDSC migration to tumor might be achieved by a variety of manipulations, including blocking of cytokines such as VEGF, G-CSF, GM-CSF, and M-CSF, as discussed above. VEGF inhibition by bevacizumab already has an established role in treatment of ovarian cancer, with approvals in both the first-line and later-line settings. Interestingly, mouse data in bevacizumab-resistant tumors demonstrated that MDSCs recruited by GM-CSF, produced by the tumor drove immunosuppression which was reversible by blockade of GM-CSF production (136). In the clinic, early phase I/II experimental approaches are focusing on blocking the receptors on MDSCs which mediate response to the above cytokines, namely CXCR2 (NCT02370238 and NCT02499328), CCR5 (NCT01736813), and CSF1R (NCT01349036). In addition, a phase 1 combination trial of the anti-CSF1R antibody cabiralizumab plus nivolumab (NCT02526017) including ovarian cancer patients is underway.

Finally, inhibition of MDSC function has been hampered to some extent by an incomplete understanding of the complex mechanisms by which MDSCs downregulate antitumor immunity. Depletion of L-arginine in the TME via expression of arginase-1 and inducible nitric oxide synthase (NOS-1) is thought to directly inhibit T cell function and result in cell cycle arrest (142). Disruption of this process may be achieved *in vitro* by inhibiting upstream inflammatory cyclooxygenase-2 (COX-2), prostaglandin E2 (PGE2) or phosphodiesterase-5 (PDE-5) signaling (143). Similarly, use of PDE-5 inhibitors such as sildenafil and tadalafil, which are already FDA approved for non-cancer indications, is being tested in combination trials in a variety of solid tumors. A phase I study of the anti-VEGFR2 molecule regorafenib and sildenafil in multiple solid tumors was reported, with evidence of disease activity including two ovarian cancer patients who achieved SD for >24 weeks (144).

## Challenges and Future Directions

Ovarian cancer remains a lethal disease due to late-stage diagnoses and a lack of suitable treatment options in the recurrent setting. Despite recent advances in PARPi and anti-VEGF treatment modalities, a better understanding of the immune cell subsets and their interactions in the peritoneal TME may bring forth novel targeted therapies and additional combination therapies. Herein, we discussed key subsets of the many immune cells that play a role in the immuno-suppressive and tumor-promoting microenvironment of ovarian cancer, and recent attempts to therapeutically employ both adaptive (**Table 2**) and innate (**Table 3**) immunity. Tumor-specific lymphocytes have been found in ovarian cancer, and have been associated with better prognoses, but anti-inflammatory cytokines produced by Tregs and other cells may overwhelm their effector functions. Depleting Tregs by cyclophosphamide has not been shown to directly affect Treg counts, but may promote overall T cell count. Reducing counts of other immunosuppressive cells, mainly those of innate immunity, may be significant in future treatments. MDSCs or M2-like macrophages may be important target populations.

**Table 2:** Published clinical trials of immunotherapies modulating adaptive immunity of ovarian cancer patients.

Intervention	NCT/Author	Phase	Enrollment	Primary Endpoint	PFS	OS	ORR
**CAR-T**							
Anti-Folate Receptor	Kershaw et al.	1	14 recurrent FR^+^ OC	Safety and Activity	–	–	*
Anti-Mesothelin	Beatty et al.	1	5 OC	Safety and Activity	–	–	0/5
**Anti-CTLA4**							
Ipilimumab	Hodi et al.	1	9 Stage 4 OC	Safety and Activity	–	–	0/9
	NCT01611558	2	40 RPS OC	DrAEs	–	–	4/39 (10.3%)
Nivolumab**+**Ipilimumab	Zamarin et al. NCT02498600	2	100 recurrent OC	ORR	3.9 months	28.1 months	16/51 (31.4%)
**Anti-PD-1**							
Nivolumab	Hamanishi et al.	2	20 RPR OC	Safety and Activity	3.5 months	20.0 months	3/20 (15%)
Nivolumab + Bevacizumab	Liu et al.	2	38 recurrent OC	ORR	12.1 months in RPS 7.7 months in RPR	–	8/20 (40%) in RPS 3/18 (16.7%) in RPR
Pembrolizumab	Varga et al.	1b	26 PD-L1^+^ OC	ORR	1.9 months	13.8 months	3/26 (11.5%)
Pembrolizumab	Matulonis et al.	2	Cohort A: 285 recurrent OC Cohort B: 91 recurrent OC	ORR	Cohort A: 2.1 months Cohort B: 2.1 months	Cohort A: - Cohort B: 17.6 months	Cohort A: 7.4% Cohort B: 9.9%
Pembrolizumab + Niraparib	Konstantinopoulos et al.	1/2	Phase 1: 9 RPR OC Phase 2: 53 RPR OC	Phase 1: Safety and RP2D Phase 2: ORR	3.4 months	–	Integrated: 18%
Durvalumab + Olaparib + Cediranib	Zimmer et al.	1	7 recurrent OC 1 peritoneal cancer 1 endomterial 1 TNBC	RP2D	–	–	4/9 (44%)
Durvalumab + Olaparib	Lampert et al.	2	35 recurrent OC	ORR	3.9 months	–	5/35 (14%)
Durvalumab + Olaparib	Drew et al.	2	32 BRCA^mut^, RPS OC	ORR	–	–	20/32 (63%)
**Anti-PD-L1**							
Avelumab	Disis et al. NCT01772004	1b	125 recurrent OC	ORR	2.6 months	11.2 months	12/125 (9.6%)

-Not reported; *No patients responded to treatment; RPR, recurrent platinum-sensitive; RPR, recurrent platinum-resistant; RP2D, Recommended phase 2 dose; TNBC, Triple-negative breast cancer; ORR, Overall response rate.

**Table 3:** Published clinical trials of immunotherapies modulating innate immunity in ovarian cancer patients.

Intervention	NCT/Author	Phase	Enrollment	Primary Endpoint	PFS	OS	ORR
**Dendritic Cell Vaccines (peptide target)**							
(Her2/neu + hTRT + PADRE) +/- cyclophosphamide	Chu et al.	1/2	14 OC in first or second remission	Safety and Activity	–	–	–
(WT p53 peptide) + IL-12	Rahma et al.	2	21 recurrent OC	Activity	8.7 months	29.6 months	–
(KLH) + IL-2	Baek et al.	1/2	10 with MRD	Safety and Activity	–	65.0 months	3/10 (30%)
(WT1)	Zhang et al.	1/2	3 OC	DrAE	–	–	0/3 (0%)
(Autologous tumor lysate) + KLH	Hernando et al.	1	6 progressive or recurrent OC	Safety and Activity	–	–	0/6 (0%)
(Autologous tumor lysate) +/- bevacizumab +/-cyclophosphamide	Tanyi et al.	1?	25 immunotherapy-naïve recurrent OC	Safety and Activity	–	–	2/25 (8%)
**Natural Killer Cells**							
NK Cells + IL-2 + CyFlu	Geller et al.	2	14 OC	Activity	–	–	4/14 (28.5%)
**Monocytes**							
**Monocytes + IFNα/γ**	Cole et al.	1	11 recurrent OC	Safety and Activity	–	–	2/11 (18.1%)

-Not reported; RPR, recurrent platinum-sensitive; RPR, recurrent platinum-resistant; RP2D, Recommended phase 2 dose; ORR, Overall response rate; KLH, Keyhole limpet haemocyanin; hTRT, human telomerase reverse transcriptase; PADRE, pan-DR epitope; WT-1, Wilms’ tumor protein 1; CyFlu, Cyclophosphamide + Fludarabine; SQ, subcutaneous; MRD, minimal residual disease.

A lack of tumor associated antigens and proper stimulation may also hinder a targeted immune response. A main problem seen in CAR-T therapies included a lack of persistence within circulation and poor penetrance into the peritoneal TME. Therapies such as checkpoint inhibitors and CAR-T cells may have demonstrated poor efficacy due to cellular interactions between adaptive and innate immunity that are yet to be fully characterized. Bregs are one class of cells that have yet to be fully understood. MDSCs have also been shown to play a significant role in decreasing immune function in the TME by secreting cytokines and directly inhibiting adaptive immune cells, but need further characterization.

Augmenting the innate immune response may be a mechanism by which to improve the anti-tumor response. Multiple groups have evaluated DCs as ways to augment a tumor-specific T cell response. Trials evaluating various peptides and whole tumor lysate have shown varied results though, but with minimal AEs. Future studies evaluating additional mechanisms of sustaining DC activation as well as possible bio-nanotechnology to replace synthesis of DCs will be critical in developing this option. Another possible mechanism is to use NK or NKT cells as an effector cell because they do not require additional activation. These cells have demonstrated the capacity to be transfected with CARs and clinical trials are currently underway. Similarly, efforts to utilize monocytes as effector cells are underway. Targeting of MDSCs is an exciting avenue for ovarian cancer therapy, with the multiple agents and combinations discussed above being tested, and many others with promising preclinical data. However, there are outstanding questions which need to be addressed in order for these approaches to be maximally beneficial. One invaluable is given the rapidly increasing number of available therapeutic combinations, how do we rationally choose which combinations to test in our patients? Ultimately, further understanding of the interactions amongst tumor and immune cells in the unique peritoneal microenvironment will allow us to better develop optimal targeted therapies for the treatment of ovarian cancer.

## Author Contributions

FN: conceived and designed the manuscript, performed the research, and wrote the manuscript. CC: wrote the manuscript and edited the manuscript. CA: conceived and designed the manuscript and edited the manuscript. All authors contributed to the article and approved the submitted version.

## Funding

Intramural Research Program, National Cancer Institute (CMA, BC011775).

## Conflict of Interest

The authors declare that the research was conducted in the absence of any commercial or financial relationships that could be construed as a potential conflict of interest.
